# Advances in modern osteotomies around the knee: Report on the Association of Sports Traumatology, Arthroscopy, Orthopaedic surgery, Rehabilitation (ASTAOR) Moscow International Osteotomy Congress 2017

**DOI:** 10.1186/s40634-019-0177-5

**Published:** 2019-02-25

**Authors:** Liang Gao, Henning Madry, Dmitrii V. Chugaev, Matteo Denti, Aleksandr Frolov, Mikhail Burtsev, Nina Magnitskaya, Victor Mukhanov, Philippe Neyret, Leonid N. Solomin, Evgeniy Sorokin, Alex E. Staubli, Kevin R. Stone, Viktor Vilenskiy, Vitaliy Zayats, Dietrich Pape, Andrey Korolev

**Affiliations:** 1 Center of Experimental Orthopaedics, Saarland University, Homburg/Saar, Germany; 2 Cartilage Net of the Greater Region, Homburg/Saar, Germany; 3 Department of Orthopaedic Surgery, Saarland University Medical Center, Homburg/Saar, Germany; 4 Vreden Russian Research Institute of Traumatology and Orthopedics, Saint-Petersburg State University, St. Petersburg, Russia; 5 Department of Knee Surgery and Sports Traumatology, IRCCS Istituto Clinico Humanitas, Rozzano, Milan, Italy; 6 People’s Friendship University of Russia, Moscow, Russia; 7 European Clinic of Sports Traumatology and Orthopaedics (ECSTO), Moscow, Russia; 8 Healthpoint, Abu Dhabi Knee & Sports Medicine Center, Zayed Sports City, Abu Dhabi, United Arab Emirates; 9 Private Clinic Sonnmatt Luzern, Lucerne, Switzerland; 10 The Stone Clinic, San Francisco, California USA; 11 Department of Bone Pathology, The Turner Scientific and Research Institute for Children’s Orthopedics, St. Petersburg, Russia; 12 Pavlov First Saint Petersburg State Medical University, St. Petersburg, Russia; 13 Department of Orthopedic Surgery, Centre Hospitalier de Luxembourg, Luxembourg, Luxembourg; 14 Sports Medicine Research Laboratory, Public Research Centre for Health, Luxembourg, Centre Médical de La Fondation Norbert Metz, Luxembourg, Luxembourg

## Abstract

Corrective lower limb osteotomies are innovative and efficient therapeutic procedures for restoring axial alignment and managing unicompartmental knee osteoarthritis. This review presents critical insights into the up-dated clinical knowledge on osteotomies for complex posttraumatic or congenital lower limb deformities with a focus on high tibial osteotomies, including a comprehensive overview of basic principles of osteotomy planning, biomechanical considerations of different implants for osteotomies and insights in specific bone deformity correction techniques. Emphasis is placed on complex cases of lower limb osteotomies associated with ligament and multiaxial instability including pediatric cases, computer-assisted navigation, external fixation for long bone deformity correction and return to sport after such osteotomies. Altogether, these advances in the experimental and clinical knowledge of complex lower limb osteotomies allow generating improved, adapted therapeutic regimens to treat congenital and acquired lower limb deformities.

## Introduction

Lower limb osteotomies are an important area in orthopaedic surgery and research, motivated largely by an increasing occurrence and distribution of osteoarthritis (OA) and other clinical problems. They are indicated for unicompartmental knee OA associated with malalignment (Madry et al. 2016), and for anatomic and functional reconstructions of posttraumatic or congenital deformities (Lamm and Paley 2004; Tetsworth and Paley 1994). Despite the unmet need for novel pharmaceutical therapies to alter the course of OA, osteotomies represent one of the very few surgical approaches to diminish the progression of the disease and to promote osteochondral repair. Particularly osteotomies around the knee such as supracondylar femoral and high tibial osteotomies (HTO) are appreciated as treatment choices for younger patients with unilateral OA, allowing for long-term preservation of the knee joint that may reduce the need for total knee replacement (TKR) (Madry et al. 2017; Pape et al. 2004; Pape et al. 2005; Pape et al. 2013a; Pape et al. 2013b; Pape and Rupp 2007), as European Arthroplasty Registers show that up to 20% of all patients with TKR are not satisfied with their knee function, mainly because of residual stiffness and pain (Kahlenberg et al. 2018; Pitto et al. 2002). As the number of osteotomies is rising, scientific interest in joint-preserving procedures is growing worldwide due to significant improvements of planning, surgical technique and implants.

To promote comprehensive discussion of complex osteotomies, more than 30 world-renowned clinicians and scientists from 16 countries, among which Abkhazia, Armenia, Austria, Belarus, France, Germany, Israel, Italy, Kazakhstan, Kirgizstan, Luxembourg, Russia, Ukraine, Switzerland, the UK, and the USA gathered together in Moscow, Russia in 2017 Congress of the Association of Sports Traumatology, Arthroscopy, Orthopaedic surgery, Rehabilitation (ASTAOR) to discuss the latest advances in complex osteotomies of the lower limb (Table 1). Reflecting the different presentations, this paper offers an overview of the key issues associated with complex osteotomies of the lower limb with a focus on high tibial osteotomies, ranging from basic scientific principles of the osteochondral unit and osteotomies to modern surgical indications, surgical planning, fixation devices, bone deformity correction techniques, paediatric osteotomies and complex surgeries, such as simultaneous osteotomy and ligament reconstruction. Certain issues regarding advanced analyses and planning knowledge for multilevel and multiplane deformities are, due to the nature of a congress report, outside of its scope and not discussed in detail.

**Table 1:** Lectures of the ASTAOR Moscow International Osteotomy Congress

Speaker	City	Country	Title of lectures
Aleksandr Artemyev	Moscow	Russia	• Twenty-year experience in correcting the leg shape and length for the aesthetic and clinical indications • Corrective osteotomy away from deformity high point for the treatment of malunited tibial fractures
Karl-Peter Benedetto	Feldkirch	Austria	Osteotomy and Instability
Dmitriy Chugaev	St. Petersburg	Russia	Errors and complications after periarticular osteotomies
Matteo Denti	Milan	Italy	• HTO: modern indications • Surgical technique of High tibial osteotomy: tips and tricks • Early OA and instability
Aleksandr Frolov	Moscow	Russia	Principles for corrective osteotomies of femur and tibia in children with osteogenesis imperfecta
Alexander Korchagin	Moscow	Russia	Rehabilitation after lower limb osteotomies
Gleb Korobushkin	Moscow	Russia	Calcaneal osteotomies in foot deformities corrections
Fedor Lazko	Moscow	Russia	Proximal tibial open wedge osteotomy with tricalciumphosphate blocks for varus tibia: easy to perform?
Oleg Milenin	Moscow	Russia	Fulkerson tibial osteotomy
Adrian Wilson	London	UK	• History of osteotomy • Complex osteotomy: intraarticular and slope change surgery • MIS surgical technique HTO and DFO • Small plates precision bone wedge and rapid rehabilitation
Henning Madry	Homburg	Germany	Update on the anatomy and pathology of the osteochondral unit Pathogenesis, definition and classification of early OA HTO and biological preservation of knee
Viktor Mukhanov	St. Petersburg	Russia	Mid-term results of high open tibial osteotomy in patients with medial compartment arthrosis
Philippe Neyret	Lyon	France	• HTO: in moderate knee arthritis • Distal femoral osteotomies • Knee osteotomies in sports
Weniamin Orljanski	Vienna	Austria	Preoperative planning for HTO
Dietrich Pape	Luxembourg	Luxembourg	• Plate fixation after HTO: which plate design is best? • Return to Sports after HTO: what to expect? • Tips and tricks for HTO: a pictorial overview
Viktor Protsko	Moscow	Russia	Distal tibial osteotomy for foot deformities correction
Evgeniy Sorokin	St. Petersburg	Russia	Chevron osteotomy of femur
Leonid Solomin	St. Petersburg	Russia	Current concepts for long bones deformities correction Is the use of orthopaedic hexapods a mainstream for the correction of long bone deformities?
Alex E. Staubli	Lucerne	Switzerland	• Fifteen years personal experience with biplanar open wedge HTO with TomoFix plate • Gap healing of open wedge HTO without interpositional grafting
Kevin R. Stone	San Francisco	USA	Meniscal replacement and grafting of the articular surfaces.
Viktor Vilenskiy	St. Petersburg	Russia	Treatment of lower limb deformities in children using different methods: external fixation, managed growth and intramedullar osteosynthesis
Igor Voronkevich	St. Petersburg	Russia	Intraarticular osteotomies for sequelar tibial condyle fractures
Vitaliy Zayats	St. Petersburg	Russia	Simultaneous ACL reconstruction and HTO

Abbreviations: ACL, anterior cruciate ligament; ASTAOR, Association of Sports Traumatology, Arthroscopy, Orthopaedic surgery, Rehabilitation; distal femoral osteotomy, DFO; HTO, high tibial osteotomy; MIS, minimally invasive surgery; OA, osteoarthritis

### A vision of osteotomy development

Recently, orthopaedic surgeons, particularly those specialized in knee surgeries and sports trauma, have noted a more frequent occurrence of early-stage OA and other symptomatic pathologies of the knee articular cartilage. Magnetic resonance imaging (MRI) explaining imaging and advancements in the field of radiology, in particular with regard to information that can be gained from weightbearing radiographs of the knee (Rosenberg et al. 1988), have made improved identification of these pathologies possible and are now routinely used (Puddu 2002). MRI scans allow visualising and classifying chondral and osteochondral knee pathologies. This is one of the reasons that the indications for an HTO have changed, together with an enhanced understanding of the effects of mechanical (un)loading on the chondrocyte, favouring unloading as a key to treat knee OA associated with axial malalignment (Waller et al. 2011).

Historically HTO was performed in cases of the late varus OA knee, the effectiveness of which was evident only for a limited number of years. This, together with advancements made on knee replacements (Nagel et al. 1996; W-Dahl et al. 2010), meant that, in the past, the HTO was progressively abandoned, and this could also be the motivation for finding new indications for the HTO procedure, as studies began to look to the HTO not for the treatment of cases of advanced OA, but for the treatment at its initial stage.

In some countries, endoprosthetic knees replaced HTOs even in young subjects, and there has been the risk of gradual loss of experience with regards to patient selection and surgical routine and which, may in turn, have negatively affected outcomes (W-Dahl et al. 2010). The situation worsens in cases of an unstable knee with ligamentous lesion of one of the cruciate ligaments or after a previously performed meniscectomy. On the other hand, some HTO studies also observed a slow deterioration over time, with good survival rates up to 15 years (Amendola and Bonasia 2010; Hui et al. 2011).

Osteotomy has traditionally been considered as a standalone treatment, but limb alignment has an intrinsic relationship with both the articulating and the stabilising structures of the knee. Therefore, it should be considered for the combination not only with other osteotomies and arthroplasties but as part of the treatment strategy whenever procedures that stabilize the knee and restore cartilage are performed.

## HTO and biological knee preservation

### Principles of biological knee preservation

There is an increasing awareness on the importance in identifying early phases of the degenerative processes in knee OA, the period of the disease when there might still be some regenerative ability of the articular cartilage, which is permanently lost in the advanced disease stages (Madry et al. 2016). A definition of the early OA phase is important to identify and properly treat patients at risk of progression, allowing to better design trials for the assessment of potential and indications of the available and new emerging treatments, and therefore to better allocate resources and manage patients affected by lesions of the knee articular surface in the clinical practice (Angele et al. 2016a; Angele et al. 2016b; Cucchiarini et al. 2016; Madry et al. 2016; Madry et al. 2012). HTO offloads the medial compartment of the knee and has been shown to promote repair of the articular cartilage damaged by OA (Jung et al. 2014; Kanamiya et al. 2002; Madry et al. 2016; Tischer et al. 2017).

Current regenerative treatment approaches for cartilage repair aim for the restoration of a focal traumatic lesion. For regenerative treatment of the knee, alignment seems also to be the most essential part, although such cases are much rarer compared to OA. Therefore, in combination with focal cartilage defect and meniscus treatments, correction of (severe) deformities is often recommended, even of smaller deformities (> 3 to 5°). A recent study showed that untreated malalignment was in 56%, graft failure in 27%, untreated meniscal deficiency in 19% and untreated instability in 5% of cases with failed index surgical procedures (Krych et al. 2018).

### Biological basis of HTO for varus deformities

In a normally aligned knee, the center of pressure passes slightly to the medial side during stance. During flexion, the center of pressure is even more medial. Axial varus malalignment leads to an abnormal load distribution across the medial compartment, pathologically affecting both tibiofemoral osteochondral units and the medial meniscus. A 4% to 6% increase in varus malalignment significantly increases loading (up to 20%) in the medial compartment, affecting the articular cartilage, the subchondral bone and the medial meniscus. HTO aims to cut through this vicious cycle of malalignment, which leads to progressive cartilage degeneration and progressive worsening of the malalignment based on the unicompartimental overload. Previous studies evaluating the effect of HTO on an intact lateral compartment in a sheep model answered the question whether the increased loads would lead to degeneration of the lateral meniscus and osteochondral unit (Ziegler et al. 2015). The lateral meniscus is potentially problematic as a prospective multicenter study has shown that patients with lateral partial meniscectomies are prone to develop rapid progressive OA within a short time (Servien et al. 2009). In a newly developed sheep osteotomy model (Pape and Madry 2013), the effects of standard and valgus overcorrection were evaluated. The increase of pressure in the lateral compartment following valgus standard correction does not lead to significant structural changes in the lateral tibiofemoral osteochondral compartment, especially no cartilage degeneration (Ziegler et al. 2015). A higher increase in load as a result of overcorrection induces a few adaptive changes in the intact lateral compartment, reflected in an increased specific bone surface in the proximal tibial subarticular spongiosa (Ziegler et al. 2015). Such valgus overcorrection also decreases the cell number in the red-red (peripheral) zone of the middle third of the lateral menisci, without changing the meniscal structure (Madry et al. 2013). These translational data are supported by several clinical studies. In the study from Bick et al., mean values of numerical meniscal parameters measured preoperatively and postoperatively by MRI (e.g. relative meniscal thickness and width) showed no significant morphological changes in either the anterior horn, pars intermedia, or posterior horn. However, when an evaluation of meniscal degeneration according to the Stoller classification with MRI images was performed, significant degeneration in every part of the meniscus [significant at the anterior horn (*p* < 0.01), pars intermedia (*p* = 0.021), and posterior horn (p < 0.01) was seen] (Bick et al. 2018). Moreover, at a mean of 2 years after medial open-wedge HTO, semiquantitative evidence of macroscopic repair of degenerated articular cartilage in the medial compartment was reported after correcting varus deformities without any additional cartilage repair procedures, especially for patients with lower body mass index (BMI) (Kim et al. 2017).

#### Modern indications for HTO

Osteotomy of the proximal tibia aims to unload the medial compartment in cases of medial tibiofemoral OA without additional surgical treatments. Degenerative changes of the articular cartilage and subchondral bone may occur through high compression or shear, and are the result of forces exerted on the bearing surfaces (Denti et al. 2013). More recently, HTO is being performed in the context of cartilage injuries, in addition to reconstructive cartilage treatments such as ACI.

The arthroscopic indication for an HTO based on medial tibiofemoral OA is when a grade 2 or 3 chondral lesion, according to the Outerbridge (Outerbridge 1961) or International Cartilage Regeneration and Joint Preservation Society (ICRS) classification (Hoemann et al. 2011), is present at the medial compartment associated with a varus knee and with an intact lateral compartment (Denti et al. 2013). The most frequent indication is seen in the young and active patient; he or she has medial pain on weightbearing, mild to moderate medial OA and deformity without bone erosion; the joint is stable and has no significant limitation of movement, and there is no patellar malposition. In this situation, a medial open-wedge osteotomy can be performed in isolation, aiming for slight overcorrection to between 0° and 6° mechanical valgus depending on the severity of initial OA changes. In recent literature, an individualized degree of correction is propagated rather than a uniform correction to the Fujisawa-Region (62–68% of the lateral tibial width) (Fujisawa et al. 1979). Fine tuning of the correction is possible intra-operatively, possibly aiming for less correction in young and active patients than older patients. The complication rate is low, although fractures through the tibial plateau and lateral cortex disruption with instability and loss of correction can occur. The procedure is often palliative, in the sense that a bone deformity will be created to compensate for intra-articular wear only, rather than correcting a pre-existing bone deformity. However, this deformity may be mild, especially when there is constitutional varus.

A guideline from the International Society of Arthroscopy, Knee Surgery and Orthopaedic Sports Medicine (ISAKOS) identified the ideal candidate for HTO as a patient with isolated medial joint line pain, aged 40 to 60 years old, BMI < 30, high-demand activity except running or jumping, malalignment < 15°, metaphyseal varus, full range of movement, and normal lateral and patellofemoral compartments (Rand and Neyret 2005). The severity of medial compartment OA is a relevant predictor of outcome after HTO, and accumulative evidences show that a low degree of OA is linked to better outcomes. Also, it is clear that tricompartimental OA is a contraindication to osteotomy (Rinonapoli et al. 1988). Correction to a femorotibial angle between 6° and 14° of valgus is associated with an optimal clinical result (Flecher et al. 2006). Undercorrection (to less than 5° of femorotibial valgus) is associated with a high (62.5%) failure rate (Rudan and Simurda 1990).

In the past, the presence of patella baja was taken as a contraindication. According to Lobenhoffer et al., the biplanar osteotomy with an V-shaped ascending frontal cut can decrease patella height by 2 mm per 10° of valgus correction (Lobenhoffer and Agneskirchner 2003). In a preexisting patella baja, the uniplanar closed wedge technique can increase patella height whereas the biplanar distal frontal technique leaves patella height unchanged. In a preexisting patella alta, either the biplanar proximal frontal open wedge technique or the obsolete uniplanar open wedge technique reduces patella alta (El-Azab et al. 2010; Hinterwimmer et al. 2011; LaPrade et al. 2010; Song et al. 2010).

An accurate technique is mandatory to obtain excellent results (Hankemeier et al. 2010; Puddu 2002; Puddu et al. 2007). The type of valgus tibial osteotomy generally indicated is an open-wedge osteotomy, in order to guarantee better and more sustainable results, this has been compared to a close-wedge osteotomy (Coventry 1965; Franco et al. 2005; Georgoulis et al. 1999; Gomoll 2011; Hernigou et al. 1987; Jackson and Waugh 1961; Puddu 2002; Puddu et al. 2007).

HTO is more recently also performed in the context of cartilage injuries in addition to reconstructive cartilage treatments (Brinkman et al. 2008)., since analyses of failed cases revealed untreated malalignment as the most commonly recognized reason (in 56% cases) for failed index surgical procedures such as microfractures or osteochondral transplantations (Krych et al. 2018). In cases of patients with focal cartilage lesions of the medial femoral condyle with accompanying varus deformities of > 5°, there is general consensus to treating such deformities either in a one- or two-staged scenario (Niemeyer et al. 2016). Data from a non-randomized controlled clinical trial on ACI in cartilage defects of the medial femoral condyle without or with HTO for patients with varus deformity of less than 5° showed that HTO also leads to a reduced rate of reinterventions and longer survival rates (Bode et al. 2013).

#### Basic principles of osteotomy planning

Multiple factors need to be considered when contemplating an HTO in each patient. Relevant issues include the age, gender and weight of the patient, their activity and expectation, and whether the patient is a smoker. The effect of excessive weight is controversial but may affect the outcome due to loss of correction, and difficulties with rehabilitation. Results of HTO tend to be better in male patients and those under 50 years, whereas the converse is true after total knee arthroplasty (Aglietti et al. 2003; Bonasia et al. 2014; Hantes et al. 2018; Keenan et al. 2018; Naudie et al. 1999; Odenbring et al. 1989). The focus should also be on location and extent of the articular wear, the origin of the deformity and joint line inclination, joint stability and motion, and leg length discrepancy.

### Patient selection

The selection of patients suitable for the HTO procedure can only be done after a thorough pre-operative evaluation. A thorough physical exam with clinical assessment of ligamentous insufficiency is prudent. The standard evaluation and deformity analysis begins with the assessment of the alignment of the lower limbs with, full leg weight bearing X-rays whose importance can not be overstated and bilateral weightbearing anteroposterior views in full extension, bilateral weightbearing posteroanterior views at 45° of flexion as described by Rosenberg (Rosenberg et al. 1988), and lateral and skyline films of both knees. The Rosenberg view has a strong predictive value when the deformity is associated with cruciate insufficiency, resulting in anterior tibial subluxation, and chondral wear prevailing in the posterior area of the medial tibial plateau. The osteotomy may be planned according to the method described by Dugdale and associates (Dugdale et al. 1992). MRI can be very useful in identifying suitable candidates for knee osteotomy, as it can show not only cartilage damage but also the stress reaction of the subchondral bone.

Before the HTO procedure, a diagnostic arthroscopy is recommended to be performed routinely in the same operative session, either for diagnostic or therapeutic purposes, allowing for determining the cartilage status to modify type/degree of correction osteotomy accordingly (Brinkman et al. 2008). Therapeutic arthroscopic procedures are also indicated in cases of intraarticular pathologies. A prospective study from Müller et al., including 340 cases of knee osteotomy, highlighted that arthroscopy prior to osteotomy around the knee as an indispensable tool with both diagnostic and therapeutic properties (Müller and Strecker 2008).

### Timing of the osteotomy

Timing is a very important factor in this reparative surgery because an osteotomy is much more effective if it is performed in the earliest stage of unicompartimental OA, in order to prevent progression of degenerative changes in the joint of a still young and active patient (Amendola and Bonasia 2010; Gomoll 2011; Trieb et al. 2006; Wolcott et al. 2010). Osteotomy is best done in cases of knees with a generally well-maintained range of motion. An osteotomy is not indicated in patients with rheumatoid arthritis, patients or in knees with greater than 20° of varus deformity. The HTO imaging indication is when the radiographs demonstrate changes such as moderate osteophytes and joint space narrowing, subchondral bone sclerosis and cysts, possible deformity of the bone contour, Kellgren-Lawrence grades II-III (Kellgren and Lawrence 1957; Luyten et al. 2012).

### Degree of articular cartilage loss

If the loss of cartilage is located postero-medial on the tibial plateau in association with an anterior cruciate ligament deficiency, a lateral closed-wedge osteotomy is logical due to the natural tendency of the procedure to decrease the tibial slope (Noyes et al. 2000). The healing time is shorter than with open-wedge osteotomy, but achieving an accurate coronal correction may be technically more challenging. Also, damage to the common peroneal nerve is possible. Controlling slope during a medial open-wedge osteotomy is more difficult, but directing the cut to the proximal tibiofibular joint will allow more freedom in adjusting the proximal fragment in the sagittal plane.

### Axial imbalance

The associated sagittal imbalance may also be treated with a biplanar osteotomy, for instance with an extension element to reduce the effects of posterior cruciate ligament deficiency, although it is important to consider the effect on the postoperative range of motion (Giffin et al. 2007). Regarding anterior instability, the isolated HTO, also known as isolated slope-reducing extension osteotomy, for the treatment of pure ACL instability is currently not an established therapeutic option with scarce available evidences (Feucht et al. 2013; Robin and Neyret 2016). However, chronic ACL or PCL instability in the presence varus osteoarthritis can be sufficiently treated with slope-decreasing (ACL insufficiency) or slope-increasing (PCL insufficiency) valgus-producing tibial osteotomies (Loia et al. 2016).

### Leg length discrepancy

Although the clinical effects of leg length discrepancy are controversial, there are significant length changes in different types of tibial osteotomy. These are rarely a problem on their own, but adding to an existing discrepancy may result in clinical significance. With a large medial open-wedge correction, the length increase may exceed 1 cm, and so a careful preoperative assessment with long X-rays and discussion with the patient are wise. Closed-wedge HTO produces a little reduction in leg length, which is less likely to be significant to the patient (Magnussen et al. 2011).

### Patella position

Patella position needs to be assessed preoperatively to exclude patellar infera (“patella baja”), as patellar height may be decreased in open-wedge osteotomy compared with closed-wedge methods. Apart from undercorrection with continued pain, and overcorrection with subsequent lateral compartment wear, patellar femoral pain is one reason for failure of the procedure, and this may be more likely with patella infera (Brouwer et al. 2005).

#### Biomechanical properties of different plates for open-wedge HTO

**Fig. 1:**
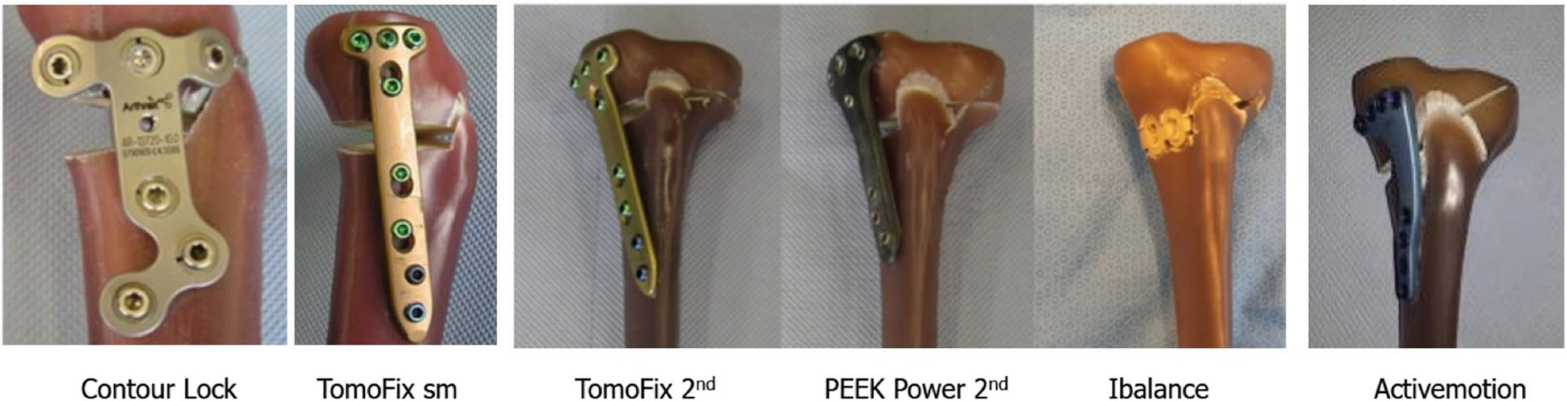
The fixation devices currently available for high tibial osteotomy, including the Contour Lock plate, TomoFix sm plate, TomoFix standard plate, PEEKPower standard plate, iBalance implant, and Activemotion plate (adapted from (Kaze et al. 2015))

A variety of different tibial osteotomy plates are currently available to perform open-wedge HTO for medial femorotibial OA in the varus knee (Lobenhoffer and Agneskirchner 2003; Pape et al. 2013b) (Fig. 1). In larger correction (< 8°), an opposite cortex fracture is frequent and inevitable, which may lead to loss of valgus correction before bony fusion is achieved and may even necessitate reoperation. In these cases, maintenance of correction depends solely on the primary implant stability prior to complete healing, therefore, sufficient primary stability until solid bone healing is crucial (Gardner et al. 2010).

**Fig. 2:**
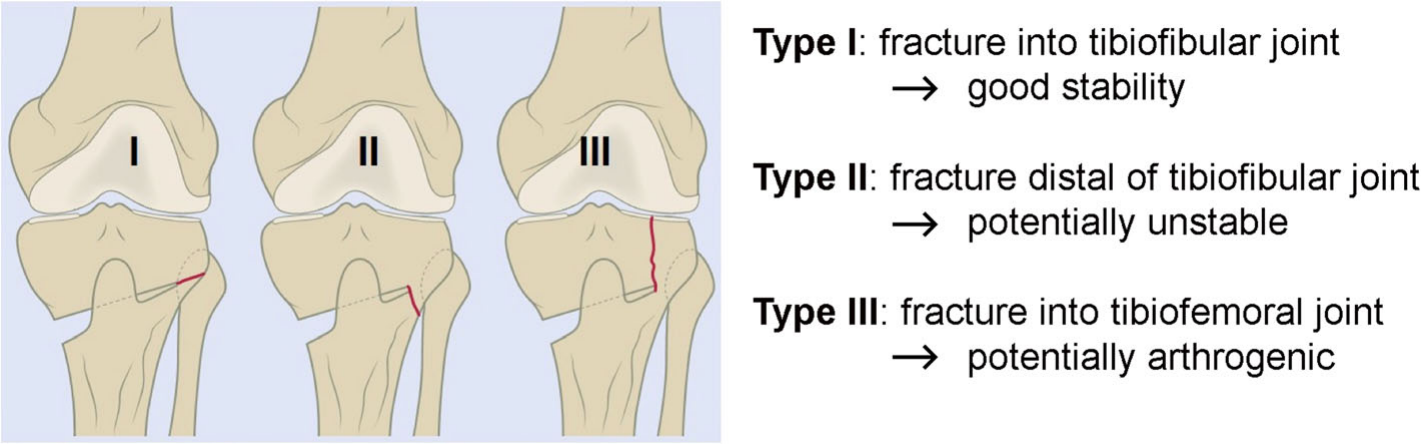
The TAKEUCHI classification of fractures around cortical hinge and corresponding stability of the subtypes (adapted from (Takeuchi et al. 2012))

Pape et al.*,* undertook a biomechanical study to compare static and fatigue strength of different plates. Twenty-six fourth-generation tibial bone composites underwent a medial open-wedge HTO according to standardized techniques, using several available implants (Maas et al. 2013). Static compression load to failure tests (Kaze et al. 2015; Maas et al. 2013) revealed that all plates showed sufficient stability (up to 2400 N) without any signs of opposite cortex fracture according to the TAKEUCHI classification (Takeuchi et al. 2012), which occurred above this load in all constructs at different load levels (Fig. 2).

During the fatigue failure tests, screw breakage in the iBalance group and opposite cortex fractures in all constructs occurred at lower loading conditions. The highest fatigue strength in terms of maximal load and number of cycles performed prior to failure was observed for the Contour Lock group followed by the iBalance implants, the TomoFix standard and small stature plates. The PEEKPower group showed the lowest fatigue strength. All plates showed sufficient stability under static loading. Compared to the TomoFix and the PEEK-Power plates, the Contour Lock plate and iBalance implant showed higher mechanical fatigue strength during cyclic fatigue testing. These data suggest that both mechanical static and fatigue strength increase with a wider proximal T-shaped plate design together with diverging proximal screws as used in the Contour Lock plate or a closed-wedge construction in the iBalance design. Mechanical strength of the bone-implant constructs decreases with a narrow T-shaped proximal end design and converging proximal screws (TomoFix) or a short vertical plate design (PEEKPower Plate). Although testing of the TomoFix plate shows not the best biomechanical results, the literature states high fusion rates and good clinical results even in corrections with wedge sizes exceeding 12 mm. A certain amount of interfragmentary motion rather than high mechanical strength and stiffness seem to be important for bone healing (Perren 2010; Staubli and Jacob 2010) and has to be studied further.

Mukhanov et al., reported the mid-term results of high open tibial osteotomy with iBalance implant, CounterLock and Puddu plate in patients with medial compartment OA. A total of 114 knees (108 patients) treated by HTO with different devices (iBalance implant, 8 knees; CounterLock, 81 knees; Puddu plate, 25 knees). Ninety-one patients (84.3%) were satisfied with the decreased pain intensity. There was no significant difference in the functional results among the three groups of patients operated with different implants. The data also confirmed that medial compartment knee OA with varus deformity, cartilage lesions with ICRS score 2–3, visual analog scale (VAS) pain intensity < 5 are an appropriate indication for HTO.

## Bone deformity correction techniques

### Prevention of lateral cortex fracture in open-wedge HTO

During the opening of a medial-based HTO, the opposite (lateral) cortex serves as an elastic hinge. In larger corrections, the capacity for elastic deformation is frequently exceeded resulting in a plastic deformation and fracture of the opposite cortex, which may lead to subsequent loss of correction (Kessler et al. 2002; Pape et al. 2004). An anteroposterior (AP) drill hole at the apex of the horizontal osteotomy (=hinge) is supposed to increase the capacity of the bony hinge for elastic deformation (Kessler et al. 2002; Schroter et al. 2014). A study investigated the possibility of the hinge drill to preserve the opposite cortex during an open-wedge HTO, comparing outcomes between HTO without or with an additional AP hinge drilling both in a Sawbone model or in human cadaveric tibial bones. Regardless of the study group, all tibial bones with an additional hinge drilling achieved larger correction angles during the spreading of the wedge until a fracture of the opposite cortex occurred. The average correction angle of all specimens without the drill hole was 2.7° and increased to 4.8° with the hinge drill (77.8% increase). The effect was pronounced in the Sawbone models (2.4° increase of correction; + 109%), while the lowest in the human tibiae (2.9° to 4.2° increase; + 45%).

The supposedly cortex-preserving effect of an anteroposterior drilling at the apex of the horizontal osteotomy of an HTO (“hinge”) increased the capacity for spreading the medial-based wedge by 2.1° with a maximum correction angle of 4.8°. In correction angles exceeding 5°, all specimens showed a hinge fracture regardless of the presence or absence of a hinge drill. These data show that the hinge protecting effect is restricted to small correction angles used i.e. to unload cartilage repair regions following microfracture, osteochondral autograft transplant and autologous chondrocyte implantation in the absence of severe malalignment. For the treatment of varus knee OA with a significant malalignment, the fracture-protecting effect of a hinge drill is absent in this study since the required angles to correct the malalignment exceed 8 ° regularly.

### Distal femoral osteotomy

Even though valgus knee deformities often have a femoral origin, distal femoral osteotomy (DFO) is an uncommonly performed procedure. There is no true algorithm to direct its use, although there are some important factors that help in decision making. DFO changes the frontal plane alignment in extension only, and any valgus deformity beyond 30° of flexion will persist. Thus in a case where there is clinical valgus and joint space narrowing in extension it will be effective, but if the joint space narrowing is only seen on the Rosenberg/weightbearing flexion view (i.e there is valgus only in flexion), it may not. In this situation, other procedures may be more appropriate. To differentiate between the origin of malalignment, separate tibial and femoral joint angles have to be measured with the mechanical lateral distal femoral angle (mLDFA, standard value 87° ± 3°) and the mechanical medial proximal tibial angle (mMPTA, standard value 87° ± 3°). If the mLDFA value is below standard, the origin of valgus deformity is the femur and a femoral osteotomy is indicated. If the mMPTA is above standard, the tibia is the origin of malalignment. If, upon reversion, the femoral angle is above standard, the femur is the origin of a varus deformity and need to be addressed accordingly.

Measuring joint line angles according to If there is valgus of mixed origin, a varus close-wedge HTO may be utilized, but can introduce joint line obliquity in this situation. Alternative options for older patients are lateral unicompartmental knee replacement or total knee arthroplasty. The ideal patient for DFO is thus a younger active patient with a valgus deformity in or near extension, with isolated lateral compartment changes or with patellar femoral symptoms. Failure of non-operative treatment, and motivation to undergo the long rehabilitation period are prerequisites.

Most published studies of DFO have concerned medial close-wedge techniques, with fewer reports of lateral opening techniques. Although the series is limited in terms of patient number and follow up, the satisfaction rates appear good, but the complication rates variable. Delayed union and non-union is an issue in both opening and closing wedge techniques, but appears to be related to surgical technique and fixation method, with higher non-union or loss of fixation rates with open-wedge osteotomy using locked plates. A study from the Centre Albert Trillat reported on 29 patients undergoing lateral open-wedge osteotomy and autologous bone grafting using an internal plate fixator, with average 80 months follow up. Eighty-six percent of patients were satisfied or very satisfied, and angular correction was good. There were 5 revisions to arthroplasty at a mean of 166 months. Most of the patients required removal of the plate due to lateral soft tissue irritation, and these were carried out at a mean of 26 months (Niemeyer et al. 2010).

The success of the technique relies on correct preoperative planning, and careful intraoperative technique. The correction is measured in a routine fashion, but including the intra-articular deformity that is due to chondral wear. If the deformity is in the metaphysis, introducing the blade parallel to the joint line will result in automatic correction as the plate is applied to the shaft proximal to the deformity. However, if the deformity is more proximally in the shaft, the blade is introduced at the appropriate angle to the joint line. The osteotomy is made after marking the bone to control rotation and preparing the blade slot. A coronal plane osteotomy anteriorly helps control the rotation and stabilize the construct intraoperatively and increases the surface area for healing. The medial cortex is weakened with multiple drill holes to allow a controlled hinged opening. This is done with osteotomies, and with the slow application of the plate to the proximal fragment as the proximal screws are inserted. Iliac crest autograft is used routinely, and the post-operative rehabilitation involves eight weeks non-weightbearing, but adapted to the clinical situation.

Lateral opening wedge distal femoral osteotomy is easier to perform, and allows more accurate angular correction, than medial closing wedge osteotomy. With careful surgical technique, adjusted to account for the origin of the deformity and aiming to correct to 0–3° varus, good functional results are achievable. Delayed union is not common, but bone grafting and protected weightbearing are required, and removal of metal must be considered routine.

### Lower limb osteotomies in children

Limb reconstruction surgery is an emerging subspecialty and corrective osteotomies are commonly applied for a wide range of indications of bone-related congenital and acquired disease (Dabis et al. 2017). Alignment correction can restore normal anatomy, physiological biomechanics, and optimal joint congruency to prevent medium and long-term degenerative deterioration of the joint.

### Limb lengthening

Current methods of limb lengthening in children are based on gradual distraction osteogenesis with various modalities (Skomoroshko et al. 2014). Vilensky et al., reported the outcomes of children long bone deformities treated with different methods (guided growth, external fixation and internal fixation). Three groups of patients were analysed: 100 children receiving osteotomies and gradual correction of deformities and lengthening by computer-assisted six axes Ortho-SUV Frame (group A),62 children receiving guided growth (group B), and 40 children receiving corrective osteotomies with internal fixation by intramedullary locking nails (group C). In group A, the accuracy of deformity correction ranged from 92% (in sagittal plane) to 96% (in frontal plane). The period of deformity correction varied from 9.18 ± 2.76 days (for simple deformities) till 24 ± 14.72 (for complex deformities). External fixation index varied from 31.2 ± 15.1 to 35.32 ± 12.61 days/cm. Complications occurred including pin-tract infection (15%), joint stiffness (17%), breakage of transosseous elements, non-union or atrophic regenerate formation (3%), and secondary fractures and deformities (4%). In group B, the accuracy of deformity correction varied from 85% (in sagittal plane) to 90% (in frontal plane). Migration of plates and screws were observed in 4 cases requiring removal and re-implantation. The guided growth was ineffective in 5 cases of Ollier disease. In group C, the accuracy of deformity correction varied from 85% (in the sagittal plane) to 90% (in the frontal plane). Peroneal neuropathy and recurrence of the deformity were observed in 1 case, respectively. The data showed that all the analyzed methods of long bone deformities treatment can be effective in children. Age, condition of growth plates, absence or presence of shortening should be taken into consideration for the treatment planning.

### Corrective osteotomies for osteogenesis imperfecta

Osteogenesis imperfecta is a connective tissue disorder characterized by low bone mass, bone fragility and long bones deformities (Marini et al. 2017). In this disease fractures of lower extremities long bones most often occur on the apex of deformities. Realignment by performing multiple closing wedge osteotomies and intramedullary nailing is a widely accepted treatment which improves patients’ mobility. Frolov and Burtsev et al., shared their experience in treatment of femur fractures and deformities with elastic titanium nails in children with osteogenesis imperfecta. Twenty-six patients (mean age 7 ± 2.5 years) with osteogenesis imperfecta underwent closing wedge osteotomies of femur on 1 or up to 3 levels and intramedullary fixation with titanium elastic nails with a postoperative immobilization from 3 to 6 weeks. At 6–8 weeks postoperatively, fracture consolidation was achieved in 77% of cases. Titanium nail migration took place in 21% cases. At 8–12 weeks postoperatively 19 patients (74%) returned to their previous level of mobility, 7 patients (26%), who were using wheelchair preoperatively, started to stand and walk with additional support. Thus, multiple closing wedge osteotomies of femur and intramedullary titanium elastic nails fixation may save or improve the level of mobility and decrease fracture incidence in children with osteogenesis imperfecta.

## Modern osteotomies

### HTO combined with cartilage repair, meniscal transplantation, and ligament reconstruction

The “modern” HTO is indicated in younger patients presenting initial medial OA of the knee and even in cases where it is associated with other surgical procedures; it not only reduces painful symptoms, but can ensure the sports activity at a recreational level (Bonnin et al. 2013; LaPrade et al. 2012; Salzmann et al. 2009; Wolcott et al. 2010). Over the last 10 years the majority of osteotomies have been performed in combined with cartilage repair, meniscal transplantation, and ligament reconstruction. For HTO associated with cartilage treatments, it has been observed that with cartilage pathologies, after a non-weightbearing period, the rate of change in the medial compartment shifts from negative to positive, indicating the potential for articular cartilage recovery secondary to an improved mechanical environment. For example, improved outcomes were observed when the HTO was accompanied with microfracture or other procedures such as autologous chondrocyte implantation (ACI) (Bode et al. 2015; Bode et al. 2013; Gao et al. 2018; Parker et al. 2011; Sterett et al. 2010; Trinh et al. 2013; Waller et al. 2011). Single-stage ACI and concomitant HTO are reliable, safe and have satisfying clinical and improved functional outcomes after 5 years (Bode et al. 2015). In the future, it will be important to compare the single-staged procedure with the two-staged HTO followed by ACI. For HTO accompanied with meniscal transplantation, the combined surgery has been reported to generate greater improvement at the final follow-up compared to isolated medial meniscal transplantation (Verdonk et al. 2006). For patients with a torn ACL, the association of HTO with ligament reconstruction is open to debate and necessitates more investigation (Denti et al. 2013).

### HTO combined with ACL reconstruction

The incidence of ACL lesions continues to increase, which is about 87 per 100,000 population per year (Nordenvall et al. 2012), with 90.1% of patients undergoing the ACL reconstruction performed arthroscopically (Paterno et al. 2014). In 56.0% of patients, surgical treatment is performed later than 6 months after injury (Razi et al. 2013), and 66.0% of the reconstruction is performed in the degenerative knee (Magnussen et al. 2013). Patients with an ACL injury present gradually complex ligamentous laxity of the knee joint with a varus malalignment and OA, which is worsened by a lateral thrust during gait. This varus thrust is a lateral joint line opening at foot strike and the patient needs to shift their weight in order to reduce the knee back into normal positioning. Three-dimensional gait analysis of patients (*n* = 33) with concomitant medial opening wedge HTO and ACL reconstruction identified a substantial decreased knee adduction in the operated limb and a slight increase in the non-operated limb within 5 years postoperatively (Marriott et al. 2015). A decrease in the knee flexion moment was also noticed at both limbs. These data suggest that such concomitant surgery yielded substantial improved ligamentous stabilization, which were maintained up to 5 years postoperatively. Therefore, similar complex pathological scenarios may necessitate such or other combined treatments. Moreover, a recent study from Keenan et al., indicated that specific factors such as older age (> 46 years), female sex, high number of comorbidities and prior meniscectomy lowered the 10-year survivorship of HTO to total knee arthroplasty, indicating that these risk factors need to be considered pre-operatively when planning intervention for isolated medial compartment OA (Keenan et al. 2018).

For patients with a failed ACL reconstruction, HTO provides a more complete solution with the inclination of the slope of the tibial plate (Won et al. 2013). In cases of anterior instability, the plate should be positioned posterior to the medial collateral ligament and allows a higher opening posteriorly than anteriorly. The combination of a valgus osteotomy with ACL reconstruction, even in cases of revision, can lead to correct axis positioning and a stable knee. This combined surgery is indicated only in young select patients, those with instability associated with initial varus OA. The results of HTO associated with ACL reconstruction are encouraging (Bonin et al. 2004; Kim et al. 2011).

### HTO with complex instability

Posterior cruciate ligament (PCL) tears are less frequent than ACL tears, which occur rarely as an isolated injury and more commonly with multidirectional instability (Musahl et al. 2015). Isolated posterior instability can be treated to increase the tibial slope by positioning the plate anteriorly. In posterior-posterolateral instability, the angle of the slope must be increased and also a valgus osteotomy must be performed with a lateral translation of the mechanical axis to 55–60% of the tibial plateau (Denti et al. 2013). If necessary, posterior cruciate ligament reconstruction can be considered at a second stage eventually in association with a posterolateral reconstruction. Gwinner et al. shows that stability after PCL reconstruction decreases over time in patients with less slope compared to patients with a normal or larger slope angle (Gwinner et al. 2017). In cases of associated anterior and posterior instability in a varus knee, HTO can be performed to correct the tibial slope for the posterior instability associated with an ACL reconstruction. It has recently been demonstrated that a moderate change of the tibial slope does not significantly modify the anterior stability (Savarese et al. 2011; Shelburne et al. 2011; Voos et al. 2012).

#### Computer-assisted navigation for osteotomies around the knee

Mechanical axis deviation of the lower extremity with knee pain and progression of clinical and radiological signs of OA of mainly single knee compartment is the indication for either distal femur or proximal tibia osteotomy. The emerging field of computer-assisted navigation aims to improve the accuracy and precision of correction angles during periarticular osteotomies of the knee by enhancing recision of preoperative planning and better control of intraoperative realignment compared with conventional knee osteotomies. The effectiveness of passive computer navigation system for osteotomies of femur and tibia in patients with posttraumatic medial knee arthritis was recently highlighted (Kornilov et al. 2015). Nine patients (mean age, 38 ± 5.6 years) with posttraumatic medial varus knee OA were operated (6 revisions). Osteotomy with a computer navigation system and plate fixation was performed at the proximal tibia and distal femur. Complications included change of the joint line and patella position, overcorrection of axis, and wrong position of tibial joint line slope. Successful bony union was observed within 1 year follow-up. The data show that guided osteotomy of femur and tibia with the computer navigation allows surgeons to precisely restore mechanical axis of lower extremity, avoid the change of knee joint line and save the right position of posterior tibial slope.

#### External fixation for long bone deformity correction

Nowadays there are different hexapod frames available for the long-bone deformity correction with reported efficiency for long bone deformity correction. Ortho-SUV Frame (OSF; Ortho-SUV Ltd., Saint-Petersburg, Russia), a novel universal unit, is composed of different types of rings without strict places for struts fixation. Solomin et al.*,* analysed the outcome of 8 cases of multilevel deformity correction using the “Spring Technique”, 5 cases of lengthening and rotation correction over the nail, 25 cases of knee joint stiffness, 13 midfoot, 11 hindfoot, and 19 complex foot deformities correction (Solomin et al. 2017). The results in the Spring Technique group were evaluated for the period of deformity correction and time in frame. In the lengthening and rotation correction group, the value of lengthening and time in frame were recorded. In the foot deformity correction group, the accuracy of deformity correction, time in frame, number and character of complications were analysed.

All cases treated with the Spring Technique obtained excellent accuracy of alignment. The following complications were reported including one case of equines deformity in ankle (treated with Achilles tendon tenonectomy and foot support) and one case of premature consolidation at one osteotomy level (treated with re-osteotomy). The average lengthening achieved in the lengthening and rotation correction group was 40.2 ± 16 mm and the time in frame was 60.7 ± 24.5 days without complications reported. In the knee joint stiffness group, the average knee joint flexion and extension after frame removal achieved were 95° and 0°, respectively. The reported complications included a pin-hole femur fracture, posterior subluxation of the lower leg, skin necrosis, instability of the frame, and deep infection (*n* = 1 each), which were treated with secondary surgeries with good final outcomes. Normal range of motion was achieved in all cases except one due to patient’s request for an early fixator removal. In the foot deformity correction group, the achieved accuracy of deformity correction was 87% for ankle, 91% for midfoot, 90% for hindfoot, and 89% for complex foot deformities. Complications reported were wire breakage (n = 1), skin necrosis (*n* = 3), pin-tract infection (*n* = 2), and preliminary consolidations (n = 3). The study shows that the Ortho-SUV Frame is an effective tool for deformity correction including flexion and extension knee stiffness, foot deformities, multilevel deformities of the long bones, and lengthening and rotation correction.

#### Return to sport after osteotomy

Evidence for the extent of sporting activities possible after valgus HTO is increasing with time; multiple studies show a significant increase in average Lysholm scores, not just in a small number of top performing patients (Robin and Neyret 2016). Those who do not return to sport may have a fear of continuing pain or progression of OA. The result of further sport on the progression of OA after HTO is not known. In the non-operated knee, recreational sport is a favourable factor and high-level sport is negative, in which it may be that HTO does not change this pattern, but in a combined alignment and stabilisation procedure, at least meniscal and chondral preservation is more likely.

The success of the surgery on return to sport depends on several variables. A recent systematic review, reporting return to sport following high tibial osteotomy in 1189 patients, indicated that approximately two-thirds (65.5%) of patients return to an equal or greater level of physical work. However, only half of competitive athletes (57%) returned to highly professional and competitive level (Ekhtiari et al. 2016). Patient motivation is central. Not surprisingly, a higher preoperative activity level is a positive predictor, usually associated with younger patients. However, the procedure is very effective in older motivated patients without instability or severe degenerative change. Whilst instability is a negative predictor for sport after isolated HTO, combined HTO and ACL reconstruction can allow it in a high percentage of cases (Pape et al. 2005). The counter to the expanding indications may be athletes’ high expectations, where they consider the goal of a normal lifestyle to include competitive sport. This must be discussed in advance of any procedure. Good rehabilitation is probably another positive factor for return to sport, although information in the literature is lacking (Lattermann and Jakob 1996). Early weightbearing using locked plates is beneficial.

In terms of the surgery, the aim of coronal correction may be different to that intended for longevity alone (Pape et al. 2013a). Whilst a variable amount of hypercorrection has been shown to be a favourable factor for HTO survival in degenerative disease, undercorrection favours return to sports, especially for those based on running. The desired correction, therefore, needs to be fine-tuned to each patient, based on their age and sporting ambitions, amongst other factors. Open-wedge correction and the use of a locked plate may be the best option, although the sagittal slope achieved will have to be borne in mind in combined injuries.

As indications broaden, patient selection will become more important (Pape and Rupp 2007). Well considered treatment algorithms in institutions that are able to continue long-term surveillance will be essential to further define the place of HTO in patients with different patterns of pathology who wish to return to sport.

## Conclusion

Corrective osteotomies around the knee are innovative and efficient therapeutic procedures for restoring axial alignment chiefly for the management of unicompartmental knee OA. This conference report presents critical insights into the up-dated clinical knowledge on osteotomies for complex posttraumatic or congenital lower limb deformities including pediatric cases, computer-assisted navigation, external fixation for long bone deformity correction and postoperative return to sport. Altogether, these advances in the experimental and clinical knowledge of complex lower limb osteotomies allow generating improved, adapted therapeutic regimens to treat congenital and acquired lower limb deformities.
